# 2-year results from an observational study of proactive treatment regimens with intravitreal aflibercept 2 mg in patients with nAMD in clinical practice: XTEND study UK cohort

**DOI:** 10.1038/s41433-024-03550-y

**Published:** 2024-12-24

**Authors:** Clare Bailey, Manju Chandran, Richard Gale, Nirodhini Narendran, James Talks, Hellen McGoey, Zinab Keshk, Peter Morgan-Warren, Helmut Allmeier, Tobias Machewitz, Praveen J. Patel, Deepali Varma

**Affiliations:** 1 Department of Ophthalmology, University Hospitals Bristol & Weston NHS Foundation Trust, Bristol, UK; 2 Department of Ophthalmology, Frimley Health NHS Foundation Trust, Frimley, UK; 3 Department of Ophthalmology, Hull York Medical School, University of York, York Teaching Hospitals NHS Foundation Trust, York, UK; 4 Department of Ophthalmology, The Royal Wolverhampton Hospitals NHS Trust NHS Foundation Trust, Wolverhampton, UK; 5 School of Life and Health Sciences, Aston University, Birmingham, UK; 6 UCL Institute of Ophthalmology, Faculty of Brain Sciences, London, UK; 7 Department of Ophthalmology, Newcastle Hospitals NHS Foundation Trust, Newcastle, UK; 8 Department of Medical Affairs, Bayer Plc, Reading, Berkshire, UK; 9 Bayer Consumer Care AG, Basel, Switzerland; 10 Bayer AG, Berlin, Germany; 11 National Institute for Health Research Biomedical Research Centre, Moorfields Eye Hospital, NHS Foundation Trust, London, UK; 12 Department of Ophthalmology, Sunderland Eye Infirmary, South Tyneside & Sunderland NHS Foundation Trust, Sunderland, UK

**Keywords:** Macular degeneration, Health care

## Abstract

**Objectives:**

The 36-month XTEND (NCT03939767) multicentre, observational, prospective study examined the effectiveness of proactive treatment regimens of intravitreal aflibercept (IVT-AFL) 2 mg in treatment-naïve patients with neovascular age-related macular degeneration (nAMD) in routine clinical practice. The 12- and 24-month outcomes from the XTEND UK cohort are reported.

**Methods:**

Patients aged ≥50 years with nAMD planned to receive IVT-AFL 2 mg were eligible. After three initial monthly IVT-AFL injections, treatment intervals could be extended in 2- to 4-weekly increments to a maximum of 16 weeks (8-week minimum treatment interval). Endpoints included mean change from baseline in best-corrected visual acuity (BCVA) and central subfield thickness (CST) at month (M) 12 and M24. Treatment intervals and safety were assessed. Statistics were descriptive.

**Results:**

In the UK, 496 patients from 23 centres were treated (mean age 79.7 years, 64.3% female). From a baseline BCVA (mean ± SD) of 55.2 ± 15.8 letters, mean (95% confidence interval [CI]) change in BCVA was +3.4 (2.0, 4.9) letters at M12 and +1.3 (− 0.3, 2.9) letters at M24. From a baseline CST (mean ± SD) of 395 ± 143 μm, mean (95% CI) change in CST was −105 ( 121, −89) μm at M12 and −105 (− 122, −88) μm at M24. By M12 and M24, patients had received a mean ± SD of 7.4 ± 2.4 and 10.7 ± 4.6 injections, respectively. Outcomes in patients enrolled prior to and during the COVID-19 pandemic were comparable. No new safety concerns were identified.

**Conclusions:**

Despite the COVID-19 pandemic, patients in the UK achieved and maintained clinically meaningful improvements in functional and anatomic outcomes through M24.

**Trial registration:**

ClinicalTrials.gov identifier, NCT03939767.

## Introduction

Neovascular age-related macular degeneration (nAMD) is the leading cause of vision loss in adults over the age of 60 years globally [1], accounting for more than half of sight-impaired and blindness certifications in the UK [2]. Anti-vascular endothelial growth factor (anti-VEGF) therapies, including intravitreal aflibercept (IVT-AFL) 2 mg, are used to treat nAMD and have demonstrated robust visual improvements in randomised clinical trials (RCTs) [3, 4]. These therapies block or inhibit VEGF, thereby reducing growth of abnormal blood vessels and leakage of existing vessels that can otherwise lead to loss of vision [5].

The RCT setting is often not representative of a real-world clinical practice. Factors including patient adherence and clinician discretion (including choice of treatment paradigm or dosing intervals) can impact treatment effectiveness [6]; patients often receive fewer injections after the first year of treatment and have shown lower visual acuity gains when compared with data from RCTs [7]. The collection of real-world evidence (RWE) is, therefore, crucial to better understand the discrepancy between outcomes observed in RCTs and clinical practice.

Previously, RWE for the effectiveness of IVT-AFL in patients with nAMD has been generated in multicentre, single-country settings: PERSEUS in Germany [8], PERSEUS-IT in Italy [9] and RAINBOW in France [10–12]. RWE in the UK has been generated previously in a retrospective electronic medical record study [13]; however, there is a need for further prospectively collected data that better reflects the current real-world clinical situation and evolving landscape.

XTEND (NCT03939767) is a multinational, multicentre, observational, prospective study examining the effectiveness of real-world proactive treatment regimens of IVT-AFL 2 mg in treatment-naïve patients with nAMD, following the broadening of the IVT-AFL label to include a flexible treat-and-extend (T&E) regimen. The study was performed in 17 countries globally, including the UK. Findings for the global cohort have been published elsewhere [14].

The XTEND study was conducted during the global COVID-19 pandemic, and treatment regimens in many countries were affected. In the UK, the Royal College of Ophthalmologists (RCOphth) released clinical guidance stating that patients must be treated at specific intervals, which greatly influenced the first year of the study (guidance was applicable in March 2020) [15].

This manuscript will report the effectiveness and safety results from the 12- and 24-month outcomes in the UK cohort of the XTEND study. This manuscript provides an opportunity to report on the real-world treatment of patients with nAMD in the UK and how clinical practice and treatment outcomes in the UK were affected by a modification to treatment regimens during the COVID-19 pandemic.

## Methods

### Study design

The XTEND study (NCT03939767) was carried out in accordance with European Medicines Agency (EMA) guidelines and regulations and any applicable local law(s) and regulation(s). Prior to the study start date, the protocol and any amendments were reviewed and approved locally by each study site’s Independent Ethics Committee or Institutional Review Board (Supplementary Table 1). Patients were enrolled into the global study between May 2019 and May 2020 and followed for 36 months.

### Patients

Treatment-naïve patients from 23 centres in the UK aged ≥50 years with nAMD were enrolled, after the decision to treat with IVT-AFL in a proactive regimen had been made as routine clinical practice, according to the locally approved label. Full inclusion and exclusion criteria are listed in the Supplementary Methods.

Only one eye per patient was included in the study. For patients where both eyes fulfilled the inclusion/exclusion criteria, the eye with the worst baseline visual acuity (VA) was included in the study. Patients were intended to be treated either by the T&E regimen or according to proactive fixed dosing (2 mg every 8 weeks). All patients provided written informed consent prior to the beginning of observations.

### Procedures

Patients received IVT-AFL 2 mg according to the local label and standard of care, at the physician’s discretion. The initial visit, first treatment, follow-up visits and end-of-observation visit took place during routine clinical practice. For the T&E regimen in the UK (EMA-aligned label), treatment intervals could be extended in 2- to 4-weekly increments to a maximum of 16 weeks. In Year 1, the minimum treatment interval was 8 weeks.

In response to the COVID-19 pandemic in the UK, RCOphth issued guidelines in March 2020 for ophthalmology clinics in the UK. These guidelines stipulated that patients with nAMD should be maintained on 8-weekly dosing unless they mentioned a significant drop in vision at their injection visit [15]. Although these guidelines were not mandatory, they were widely adopted in UK clinics. This treatment regimen modification in response to the pandemic led to alterations to planned proactive treatment regimens. This guidance, provided in response to the pandemic, was reflected in recommendations issued for physicians internationally [16].

### COVID-19 sensitivity analyses

As the COVID-19 pandemic began after the study start date, COVID sensitivity analyses were performed. In the global cohort, the ‘pre-COVID’ group included all patients who received their regular end-of-observation visit before the start date of the COVID-19 pandemic in their country of residence, or who received their first injection 180 days prior to the start date of the COVID-19 pandemic in their country of residence. The ‘during COVID’ group included all other patients. The pandemic start date was provided by Bayer representatives and was based on individual national guidelines (March 2020 in the UK).

### Study endpoints and analyses

The primary efficacy endpoint was mean change from baseline in best-corrected visual acuity (BCVA) at Month 12. The preferred method of measuring BCVA was by Early Treatment Diabetic Retinopathy Study (ETDRS) letters or Snellen chart with conversion to ETDRS.

Secondary endpoints included from baseline: change in BCVA at 24 months; change in BCVA by intended treatment regimen at 12 and 24 months; proportion of patients with predetermined visual gains and losses (equivalent to 5, 10 and 15 ETDRS letters) at 12 and 24 months; proportion of patients achieving a Snellen equivalent of 20/40 or better at 12 and 24 months; change in central subfield thickness (CST) measured using spectral-domain optical coherence tomography at 12 and 24 months; and injection number.

Patients who received ≥1 IVT-AFL injection were included in the safety analysis set (SAS); those who received ≥1 IVT-AFL injection, had at least one VA measurement with available BCVA letter score at baseline and at least one post-baseline assessment valid for analysis (i.e. measured ≥5 days after an injection) were included in the full analysis set (FAS). VA and CST outcomes were evaluated at baseline and monthly until Month 24. The 12-month completers were defined as those who had a valid VA assessment at baseline and at Month 12 (360 ± 60 days).

### Safety

Safety was assessed throughout the study period. All adverse events (AEs) were reported and coded using the Medical Dictionary for Regulatory Activities system. Treatment-emergent AEs (TEAEs) were defined as those that occurred after the first IVT-AFL injection and within 30 days after the last injection. Safety analyses were performed on TEAEs; AEs that were not treatment-emergent were listed without further analysis.

### Statistical analysis

Statistical analyses were explorative and there were no pre-defined hypotheses. All variables were analysed descriptively: categorical variables by frequency tables and continuous variables by sample statistics (i.e. mean, standard deviation, minimum, median, quartiles and maximum). Continuous variables are described by absolute value and as change from baseline per analysis time point, if applicable. For missing data in BCVA or CST, last observation carried forward (LOCF) was used.

## Results

### Patients

**Fig. 1:**
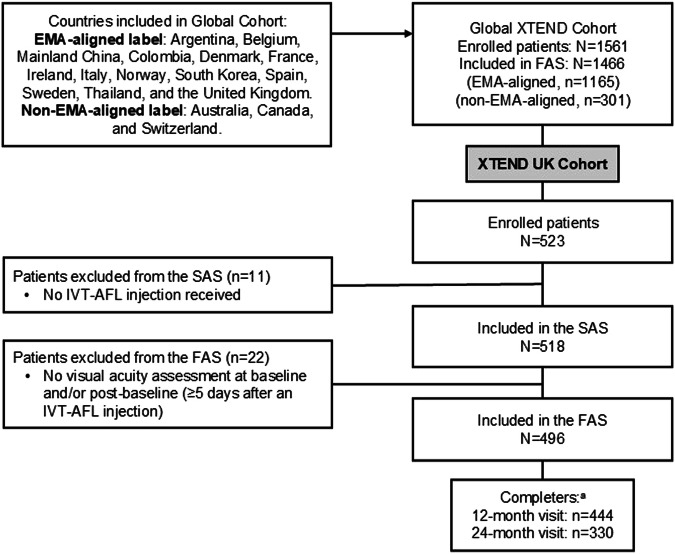
Patient disposition. ^a^Patients with a visit within the 12-month (360 ± 60 days) or 24-month (720 ± 60 days) visit window. EMA, European Medicines Agency; FAS, full analysis set; IVT-AFL, intravitreal aflibercept; SAS, safety analysis set.

Globally, XTEND enrolled 1561 patients from 127 centres. Of these, 523 patients from 23 centres in the UK were enrolled, of which 518 patients comprised the SAS. A total of 496 patients were included in the FAS; 22 patients were excluded from the FAS as there was no VA assessment at baseline and/or post-baseline visit (Fig. 1).

The mean age of patients in the XTEND UK cohort was 79.7 years, and 64.3% were female (Table 1). At baseline, the mean ± SD BCVA was 55.2 ± 15.8 letters and 22.8% of patients had a baseline BCVA of ≥70 letters. At baseline, mean ± SD CST was 395 ± 143 µm (Table 1). The majority of patients were intended to be treated according to a proactive T&E regimen (76.6%); the remaining patients (23.4%) were intended to be treated according to a proactive fixed-dosing regimen.

**Table 1:** Patient baseline demographics and disease characteristics (FAS).

Characteristic	XTEND UK Cohort
**Number of patients**	496 (100.0)
**Mean age, years±SD**	79.7±8.1
**Sex, n (%)**	
Female	319 (64.3)
**Race, n (%)**	
White	461 (92.9)
Black or African American	2 (0.4)
Asian	4 (0.8)
Indigenous	1 (0.2)
Mixed ancestry	18 (1.5)
Not reported	28 (5.6)
**Mean BCVA, ETDRS letters±SD**	55.2±15.8
**Mean CST, µm±SD**	395±143
**BCVA letter score category, n (%)**	
<35	44 (8.9)
≥35 to <70	339 (68.3)
≥70	113 (22.8)
**Primary intended treatment regimen after initial monthly injections, n (%)**	
Proactive fixed treatment	116 (23.4)
Proactive treat-and-extend	380 (76.6)

*BCVA* best-corrected visual acuity, *CST* central subfield thickness, *ETDRS* Early Treatment Diabetes Retinopathy Study, *FAS* full analysis set, *SD* standard deviation.

### Functional outcomes

**Fig. 2:**
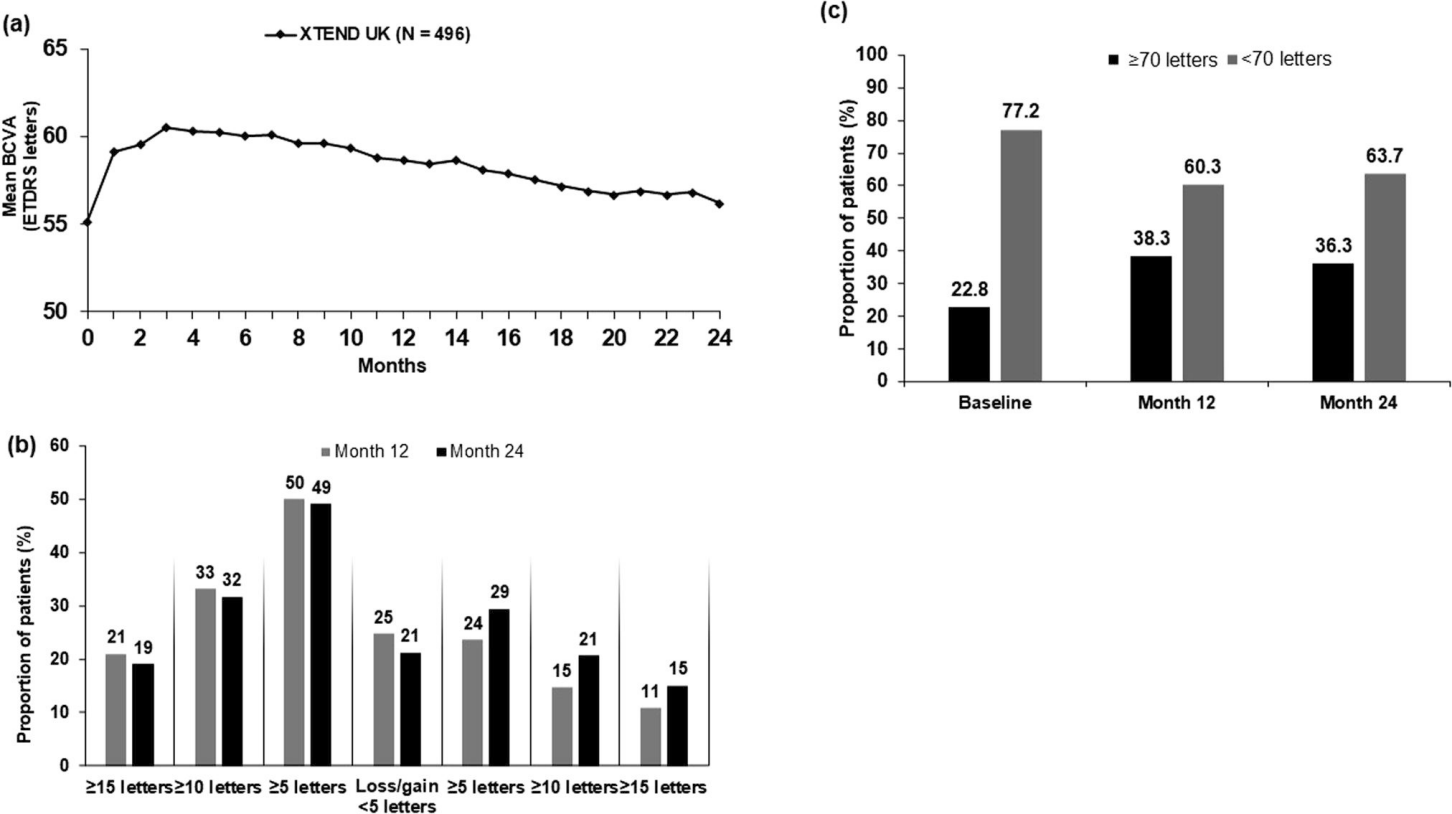
Visual acuity outcomes for patients with treatment-naïve nAMD who were treated with IVT-AFL in routine clinical practice. **a** Mean absolute BCVA over 24 months. **b** Proportion of patients stratified by BCVA categorical score change over 24 months. **c** Proportion of patients achieving ≥70 or <70 letters at baseline, Month 12 and Month 24 (Snellen equivalent of 20/40). FAS, LOCF. In (**a**), the mean BCVA data are based on the nearest VA assessment within the monthly +/-15-day visit window. BCVA best-corrected visual acuity, ETDRS Early Treatment Diabetic Retinopathy Study, FAS full analysis set, IVT-AFL intravitreal aflibercept, LOCF last observation carried forward, nAMD neovascular age-related macular degeneration; VA visual acuity.

For patients in the XTEND UK FAS, the mean (95% confidence intervals [CIs]) change in BCVA from baseline was +3.4 (2.0, 4.9) letters at Month 12 and +1.3 (− 0.3, 2.9) letters at Month 24 (Table 2, Fig. 2a and Supplementary Fig. 1a). When the mean change in BCVA was stratified by baseline BCVA, the change in BCVA was numerically highest at Months 12 and 24 in patients with a baseline BCVA of <35 letters (+ 10.5 at Month 12 and +6.6 at Month 24). In patients with a baseline BCVA of ≥35 to <70 letters, the change in BCVA was +3.8 at Month 12 and +1.7 letters at Month 24. In patients with a baseline BCVA ≥ 70 letters, BCVA was maintained above 70 letters (− 0.3 letters at Month 12 and −2.1 letters at Month 24) (Supplementary Fig. 1b).

**Table 2:** Mean change in visual acuity (letters) from baseline to Months 12 and 24 (FAS; LOCF and completers).

	XTEND UK Cohort	
	FAS (LOCF) (N=496)	FAS (Completers^a^) (n=356)
**Baseline**		79.7±8.1
Mean BCVA	55.2±15.8	55.6±15.6
n, %	496 (100.0)	356 (100.0)
**Baseline of patients with 24-month BCVA**		
Mean BCVA	–	55.9±15.4
n, %	–	293 (82.3)
**Month 12**		
Mean BCVA	58.7±19.8	60.1±18.4
Change from BL	3.4 (2.0, 4.9)	4.6 (3.0, 6.2)
n, %	489 (98.6)	356 (100.0)
**Month 24**		
Mean BCVA	56.5±21.6	59.0±18.7
Change from BL	1.3 (−0.3, 2.9)	3.2 (1.3, 5.0)
n, %	496 (100.0)	293 (82.3)

*BCVA* best-corrected visual acuity, *BL* baseline, *CI* confidence interval, *FAS* full analysis set, *LOCF* last observation carried forward.

Data are mean ± SD and mean (95% CI) unless otherwise stated.

^a^Completers defined as patients who had valid visual acuity assessments at baseline and at Month 12 (360 ± 60 days).

The proportion of patients with treatment-naïve nAMD who achieved ≥5-letter, ≥10-letter and ≥15-letter BCVA gains by Month 24 were 49.4%, 31.7% and 19.2%, respectively (Fig. 2b). Overall, 84.9% of patients maintained vision (i.e. lost fewer than 15 letters) from baseline to Month 24, and there were no marked differences between the letter gains and losses by Month 12 and Month 24 (Fig. 2b). The proportion of patients with a BCVA of ≥70 letters (approximately 20/40 Snellen equivalent) in the study eye increased from 22.8% (113/496) at baseline to 36.3% (180/496) after Month 24 (Fig. 2c).

### Anatomic outcomes

In the XTEND UK FAS, from a baseline CST (mean ± SD) of 395 ± 143 μm, mean (95% CI) change in CST was −105 (− 121, −89) μm at Month 12 and −105 (− 122, −88) μm at Month 24 (Supplementary Fig. 2 and Supplementary Table 2).

### Treatment pattern and exposure

**Fig. 3:**
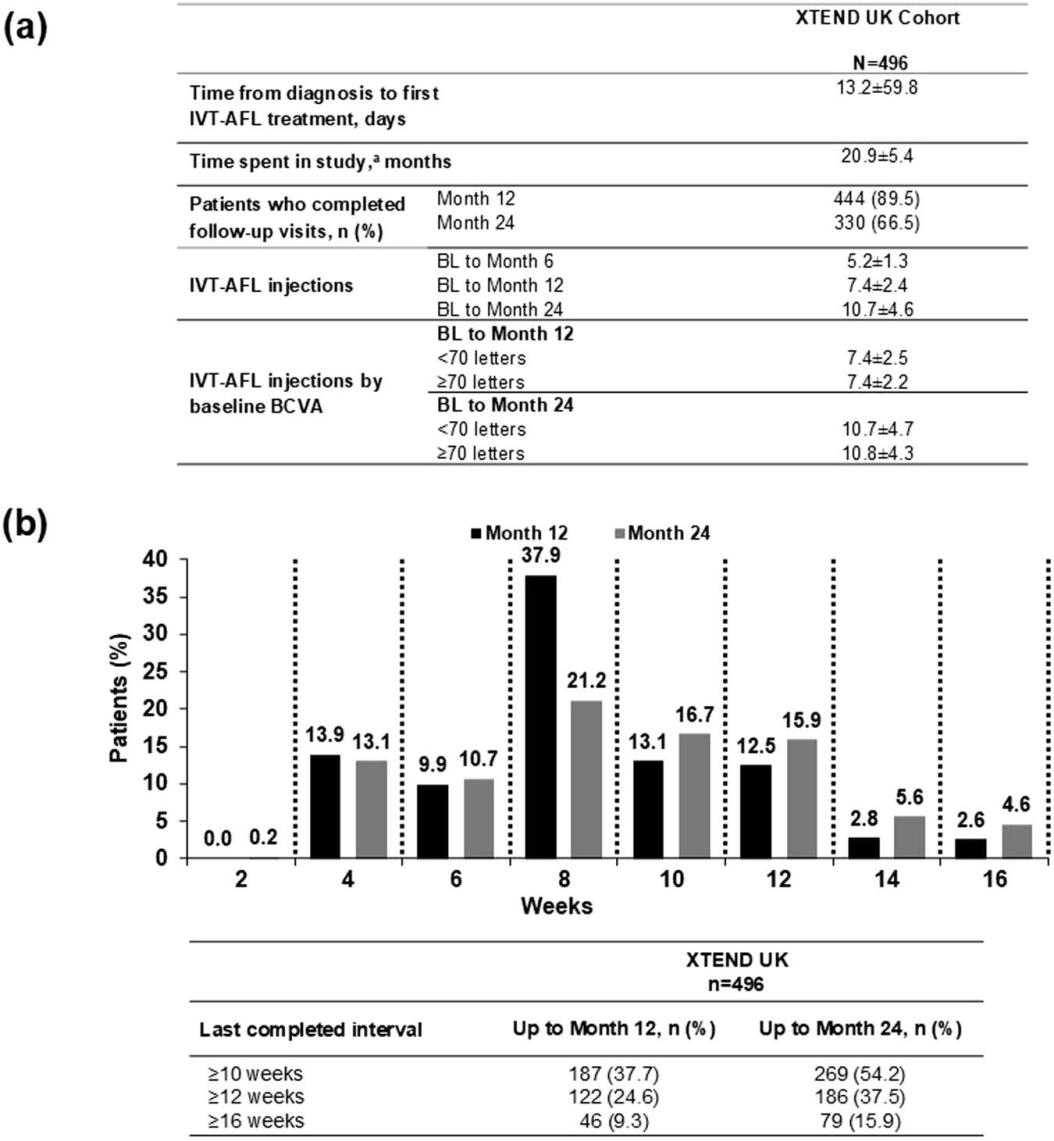
Treatment patterns. **a** Mean time from diagnosis to first IVT-AFL treatment, mean time in study and mean number of IVT-AFL treatments from baseline to Month 12 and 24 in the study eye. **b** Last injection interval by Month 12 and Month 24. FAS, OC. Values are mean ± SD unless otherwise stated. Data was missing for three patients at Months 12 and 24. ^a^Defined as the mean time between first and last IVT-AFL injections. BL baseline, FAS full analysis set, IVT-AFL intravitreal aflibercept, OC observed completers, SD standard deviation.

Mean ± SD time from diagnosis to first IVT-AFL injection was 13.2 ± 59.8 days (median [interquartile range]: 2.0 [1.0, 11.0] days), and average time in the study was 20.9 ± 5.4 months (Fig. 3a). In total, patients received a mean ± SD of 5.2 ± 1.3 injections between baseline and Month 6, 7.4 ± 2.4 injections between baseline and Month 12, and 10.7 ± 4.6 injections between baseline and Month 24 (Fig. 3a).

The last completed treatment interval was ≥12 weeks in 24.6% (122/496) of patients at Month 12, and 37.5% (186/496) at Month 24, and ≥16 weeks in 9.3% (46/496) of patients at Month 12, and 15.9% (79/496) at Month 24 (Fig. 3b).

### Impact of the COVID-19 pandemic and UK RCOphth guidance

In the XTEND UK cohort, the COVID-19 pandemic had minimal impact on key functional and anatomic endpoints, when comparing patients with treatment-naïve nAMD who began treatment before or during the pandemic.

From an overall similar mean baseline BCVA of 55.0 ± 16.0 letters and 55.4 ± 22.4 letters in the ‘pre-COVID’ group and ‘during COVID’ group, respectively, at 24 months, the mean (95% CI) change in BCVA was similar in patients who started treatment before the COVID-19 pandemic (n = 204; +1.3 [− 1.1, 3.8] letters) and patients who started treatment during the pandemic (n = 292; +1.2 [− 0.9, 3.3] letters).

The mean ± SD CST change at 24 months was similar in patients who started treatment during the COVID-19 pandemic (n = 207; −106 ± 165 µm [baseline: 375 ± 122 µm]) and patients who started treatment before the COVID-19 pandemic (n = 122; −103 ± 133 µm [baseline: 407 ± 153 µm]).

Before and during the COVID-19 pandemic, observed completers received a mean ± SD of 5.6 ± 1.2 (n = 139) and 5.4 ± 1.2 (n = 217) injections between baseline and Month 6, respectively, 8.1 ± 2.4 and 8.0 ± 2.0 injections between baseline and Month 12, respectively, and 11.8 ± 4.5 and 11.9 ± 4.0 injections between baseline and Month 24, respectively.

### Safety

In the XTEND UK cohort, TEAEs were reported in 37.1% (192/518) of patients (Supplementary Table 3). In the study eye, 85 patients (16.4%) reported ocular TEAEs; the most common ocular TEAEs (grouped using MedDRA preferred terms) in the study eye (defined as an incidence ≥1%) were cataract (17/518; 3.3%), blepharitis (9/518; 1.7%), cataract nuclear (7/518; 1.4%), conjunctival haemorrhage (6/518; 1.2%) and visual impairment (5/518; 1.0%).

Overall, five cases of intraocular inflammation were reported in the study eye, defined by the following preferred terms: eye infection (n = 3), endophthalmitis (n = 1) and iridocyclitis (n = 1). No cases of retinal vasculitis, retinal occlusive vasculitis and retinal artery occlusion were reported in the cohort. In total, four deaths were reported in this cohort; three of which were reported as treatment-emergent.

## Discussion

XTEND is the largest real-world study to date to prospectively evaluate patients with nAMD treated with IVT-AFL 2 mg. The functional and anatomic improvements observed in the 12- and 24-month analysis of the UK cohort demonstrated the effectiveness of IVT-AFL in patients with treatment-naïve nAMD in routine clinical practice.

In this UK patient population, mean change in BCVA from baseline was +3.4 at Month 12 and +1.3 at Month 24, with the greatest improvement achieved in patients with a baseline BCVA of <35 letters ( + 6.6 letters). The overall change from baseline is lower than that observed in the global XTEND population ( + 4.3 and +2.3 letters by Month 12 and Month 24, respectively) [14] and the VIEW RCT ( + 7.6 letters by Week 96) [17]. Patients with a high baseline BCVA ( ≥ 70 letters) maintained vision over 24 months, and the number of patients with ≥70 letters (20/40 Snellen equivalent) increased from 22.8% at baseline to 36.3% at Month 24. This is of particular interest as above 70 letters is the required VA in order to legally drive in the UK [18], enabling patients to gain independence and, in turn, improve their quality of life [19].

This study showed an improvement in CST from baseline (− 105 µm and −105 µm at Month 12 and Month 24, respectively). This CST improvement was similar to that observed in the global XTEND cohort (–106 μm and –109 μm at Months 12 and 24, respectively) [14]; however, the improvement was not as pronounced as observed in some RCTs (–128 μm at Week 96 in the VIEW study [17]). This may be related to patients receiving fewer injections in the XTEND UK cohort (7.4 and 10.7 injections by Month 12 and Month 24, respectively) compared with the number of injections observed in RCTs (11.2 injections at Week 96 in VIEW) [17]. However, the number of injections observed in this XTEND UK cohort was similar to other observational studies of IVT-AFL in nAMD in Europe [8–10].

The aforementioned visual gains in the XTEND UK cohort were numerically lower than those observed in the XTEND global cohort (UK, +3.4 ± 16.9 vs Global, +4.3 ± 17.6 letters at Month 12), despite a comparable baseline VA (UK, 55.2 ± 15.8 vs Global, 54.3 ± 20.3) and fewer patients entering the study with a high VA ( ≥ 70 letters [UK, 22.8% vs Global, 28.1%]) [14].

Lower VA gains in the UK cohort may be attributed to a slightly older population in the UK cohort than in the Global cohort (UK, 79.7 years vs Global, 78.5 years), but could also be attributed to guidance shared by RCOphth in March 2020, which was limited to the UK; the guidance stipulated that patients with nAMD should be maintained on 8-weekly anti-VEGF injections with no clinical review (unless there was a significant loss in vision since the previous visit) [15]. This resulted in most patients receiving IVT-AFL with an injection interval of 8 weeks; at Month 12 and Month 24, 37.9% and 21.2% of patients had a last injection interval of 8 weeks, respectively. Studies conducted prior to the COVID-19 pandemic evaluating a T&E regimen reported a last treatment interval of 8 weeks at Month 12 in up to 41.5% and 59.5% of patients in the clinical trial and real-world settings, respectively. At Month 24, fewer patients in the UK cohort had an injection interval shorter than 8 weeks when compared with the Global cohort (UK, 24.0% vs Global, 30.4%) [14]; this is also likely due to the RCOphth guidelines described above. While the number of injections administered in the UK and Global cohorts were similar (UK, 7.4 ± 2.4; Global, 7.7 ± 2.7 at Month 12), the strict COVID-19 restrictions imposed by the RCOphth reduced the flexibility normally afforded to physicians to adjust treatment intervals per patient needs. It is for this reason the T&E treatment paradigm may be preferred in routine clinical practice, as it allows the clinician to adjust treatment according to the needs of the patient, thereby, reducing treatment burden whilst achieving and maintaining robust visual gains [6]. The potential impact of the RCOphth guidelines during COVID on VA gains and injection intervals is speculative and a post-hoc analysis of treatment patterns would be required in order to verify this hypothesis. Despite the influence of COVID-19 restrictions on IVT-AFL treatment, functional and anatomic improvements were achieved at Month 12 and maintained up to Month 24, and mean injection number before and during the COVID pandemic were similar. The impact of the COVID-19 pandemic on the treatment of nAMD has been evaluated in other countries, with variable findings, which may be reflective of the differences in restrictions imposed. An observational study from the FRB! Database, which included eight countries internationally, reported a loss in vision during the COVID-19 period. The mean change in BCVA from 6 months before to 6 months’ post-lockdown ranged from –0.4 to –3.8 letters across the countries evaluated, and this reduction was in proportion with a reduced number of injections [20].

In our study, for the T&E regimen in the UK (EMA-aligned label), treatment intervals could be extended in 2- to 4-weekly increments to a maximum of 16 weeks. However, findings show that the label was not strictly followed, and at Months 12 and 24, approximately 6.7% and 11.3% of patients, respectively, had treatment intervals of longer than 16 weeks. This may be reflective of decisions taken in routine clinical practice, an inherent aspect of real-world research, or related to the impact of COVID-19. As expected, the proportion of patients with treatment intervals beyond 12 weeks in the UK XTEND cohort (24.6% at Month 12 and 37.5% at Month 24) were lower than those observed in the clinical trial setting; treatment extensions beyond 12 weeks at Month 12 have been reported in patients with nAMD treated with aflibercept 2 mg in the ALTAIR trial, where up to 40.5% of patients achieved an extended injection interval of 16 weeks at Week 52 [21].

The safety profile of IVT-AFL in the UK over 24 months was consistent with prior observational studies [5, 8, 10, 17]. Four deaths were reported in the XTEND UK cohort during the prevailing COVID-19 pandemic.

The strengths of the XTEND study include its real-world, prospective design, long study duration and large patient population that is reflective of patients with nAMD. The multicentre design allowed for data collection in a variety of real-world clinical settings. Importantly, the XTEND study provided an opportunity to reflect on treatment outcomes, following modifications to treatment regimens during the COVID-19 pandemic.

There are limitations to the XTEND study inherent to the observational, prospective design, including the non-controlled design, lack of randomisation and a heterogenous population. Information bias may have been introduced in this UK population due to the various methodologies used to assess VA; in XTEND, BCVA was the preferred method, but where that was not available (or is not standard practice), VA was accepted as best measured. In addition, all prescribing decisions were made at the discretion of the treating clinician, which can result in diverse treatment patterns and, therefore, variability in treatment outcomes; however, prescribing decisions in the XTEND study were made by clinicians with extensive clinical experience. Moreover, as a real-world study, there were a number of non-completers and missing data in the XTEND UK cohort, although these numbers were in line with what is expected in RWE studies [9, 10].

In conclusion, IVT-AFL proactive treatment regimens (either fixed or T&E) were found to be a robust treatment option for treatment-naïve patients with nAMD in the UK. Despite the COVID-19 pandemic, clinically meaningful improvements in functional and anatomic outcomes were achieved by Month 12 and maintained through Month 24, with similar injection numbers before and during the pandemic, indicating that a proactive regimen of IVT-AFL 2 mg proactive regimen can offer improved visual outcomes under real-world conditions.

Supplemental material is available at Eye’s website

## Summary

### What was known before:

- Neovascular age-related macular degeneration (nAMD) accounts for more than half of sight-impaired and blindness certifications in the UK.
- Anti-vascular endothelial growth factor therapies, such as intravitreal aflibercept (IVT-AFL), are a robust treatment option for nAMD; however, poorer visual outcomes are achieved in routine clinical practice compared with randomised clinical trials.

### What this study adds:

- The XTEND study gathered real-world evidence from patients with treatment-naïve nAMD who were treated with a proactive IVT-AFL treatment regimen (fixed dosing or treat-and-extend) in the UK.
- Despite the influence of COVID-19 restrictions on IVT-AFL treatment, an IVT-AFL proactive regimen was found to be a robust treatment option for patients with treatment-naïve nAMD in the UK.

## Supplementary information

XTEND UK 2-year supplement

## Data Availability

Availability of the data underlying this publication will be determined later according to Bayer’s commitment to the European Federation of Pharmaceutical Industries and Associations/Pharmaceutical Research and Manufacturers of America “Principles for responsible clinical trial data sharing.” This pertains to scope, time point, and process of data access. As such, Bayer commits to sharing, upon request from qualified scientific and medical researchers, patient-level clinical trial data, study-level clinical trial data, and protocols from clinical trials in patients for medicines and indications approved in the United States (US) and European Union (EU) as necessary for conducting legitimate research. This applies to data on new medicines and indications that have been approved by the EU and US regulatory agencies on or after January 1, 2014. Interested researchers can use www.clinicalstudydatarequest.com to request access to anonymized patient-level data and supporting documents from clinical studies to conduct further re-search that can help advance medical science or improve patient care. Information on the Bayer criteria for listing studies and other relevant information is provided in the ‘Study sponsors’ section of the portal. Data access will be granted to anonymized patient-level data, protocols, and clinical study reports after approval by an independent scientific review panel. Bayer is not involved in the decisions made by the independent review panel. Bayer will take all necessary measures to ensure that patient privacy is safeguarded.
