# Supplementary material for: 2-year results from an observational study of proactive treatment regimens with intravitreal aflibercept 2 mg in patients with nAMD in clinical practice: XTEND study UK cohort
Source: Eye (Lond). 2024 Dec 24;39(6):1138–45. doi: 10.1038/s41433-024-03550-y (PMC11978859; doi:10.1038/s41433-024-03550-y)
Supplement: Supplementary file 1 — XTEND UK 2-year supplement [file 41433_2024_3550_MOESM1_ESM.docx]

## SUPPLEMENTARY MATERIALS

## Bailey et al. 2-year results from an observational study of proactive treatment regimens with intravitreal aflibercept 2 mg in patients with nAMD in clinical practice: XTEND study UK cohort

## Supplementary Item 1: Supplementary methods.

## Supplementary Table 1: Ethics approval boards.

## Supplementary Fig. 1: Change in visual acuity in patients with treatment-naïve nAMD who received IVT-AFL in routine clinical practice.

## Supplementary Table 2: Mean change in CST measured by OCT (μm) from baseline to Month 12 and Month 24 in patients with treatment-naïve nAMD who received IVT-AFL in routine clinical practice.

## Supplementary Fig. 2: Mean change in CST measured by OCT (μm) from baseline to Month 12 and Month 24 in patients with treatment-naïve nAMD who received IVT-AFL in routine clinical practice.

## Supplementary Table 3: Safety overview cumulative up to 24 months (SAS).

**Supplementary Methods**

- Inclusion criteria: nAMD diagnosis; decision to initiate IVT-AFL treatment in a proactive regimen made as part of routine clinical practice; treatment-naïve in the study eye;
  ≥50 years of age; and informed written consent.
- Exclusion criteria: Participation in an investigational programme with interventions outside of routine clinical practice; contraindications to IVT-AFL listed in local marketing authorisation; planned treatment regimen outside of local marketing authorisation; eye diseases in the study eye likely to require surgery during the observation period; concomitant ocular or systemic administration of drugs ≤3 months before IVT-AFL treatment that could interfere with or potentiation the mechanism of action of IVT-AFL; and any other retinal disease.
- The global XTEND study included an overall enrollment target of ≥2000 patients, including ≥1200 patients in EMA-aligned countries, such as the UK, as determined by feasibility and described by Korobelnik et al (1).

**Supplementary Table 1: Ethics approval boards.**

| **Country** | **Site number** | **Site name** | **Name of ethics committees** | **Number/ID of the approvals** |
| --- | --- | --- | --- | --- |
| United Kingdom | 12001 | Bristol Eye Hospital | South Central - Oxford C Research Ethics Committee and HRA and Health and Care Research Wales (HCRW) Approval | 19/SC/0148 |
| United Kingdom | 12002 | Queen Alexandra Hospital |  |  |
| United Kingdom | 12003 | Leicester Royal Infirmary |  |  |
| United Kingdom | 12004 | King's College Hospital |  |  |
| United Kingdom | 12005 | Colchester Eye Centre |  |  |
| United Kingdom | 12006 | Singleton Hospital |  |  |
| United Kingdom | 12007 | Stoke Mandeville Hospital |  |  |
| United Kingdom | 12008 | Westmorland General Hospital |  |  |
| United Kingdom | 12009 | Royal Victoria Infirmary |  |  |
| United Kingdom | 12010 | Gloucestershire Royal Hospital |  |  |
| United Kingdom | 12011 | Aberdeen Royal Infirmary |  |  |
| United Kingdom | 12012 | Royal Liverpool University |  |  |
| United Kingdom | 12013 | Norfolk and Norwich University Hospital |  |  |
| United Kingdom | 12014 | Royal Victoria Hospital |  |  |
| United Kingdom | 12015 | The York Hospital |  |  |
| United Kingdom | 12016 | Sunderland Eye Infirmary |  |  |
| United Kingdom | 12017 | Birmingham & Midland Eye Centre |  |  |
| United Kingdom | 12018 | Frimley Park Hospital |  |  |
| United Kingdom | 12019 | New Cross Hospital |  |  |
| United Kingdom | 12020 | Moorfields Eye Hospital |  |  |
| United Kingdom | 12021 | East Kent Hospitals |  |  |
| United Kingdom | 12022 | Peterborough City Hospital |  |  |
| United Kingdom | 12023 | Epsom and St Helier Hospital |  |  |
| United Kingdom | 12024^a^ | Southampton Hospitals |  |  |

^a^Ethics approval was received; however, the site did not open to recruitment.

**Supplementary Fig. 1: Change in visual acuity in patients with treatment-naïve nAMD who received IVT-AFL in routine clinical practice.** (**a**) Mean change in visual acuity over 24 months. (**b**) Mean change in visual acuity from baseline to Months 12 and 24 stratified by baseline visual acuity.

**(a)**


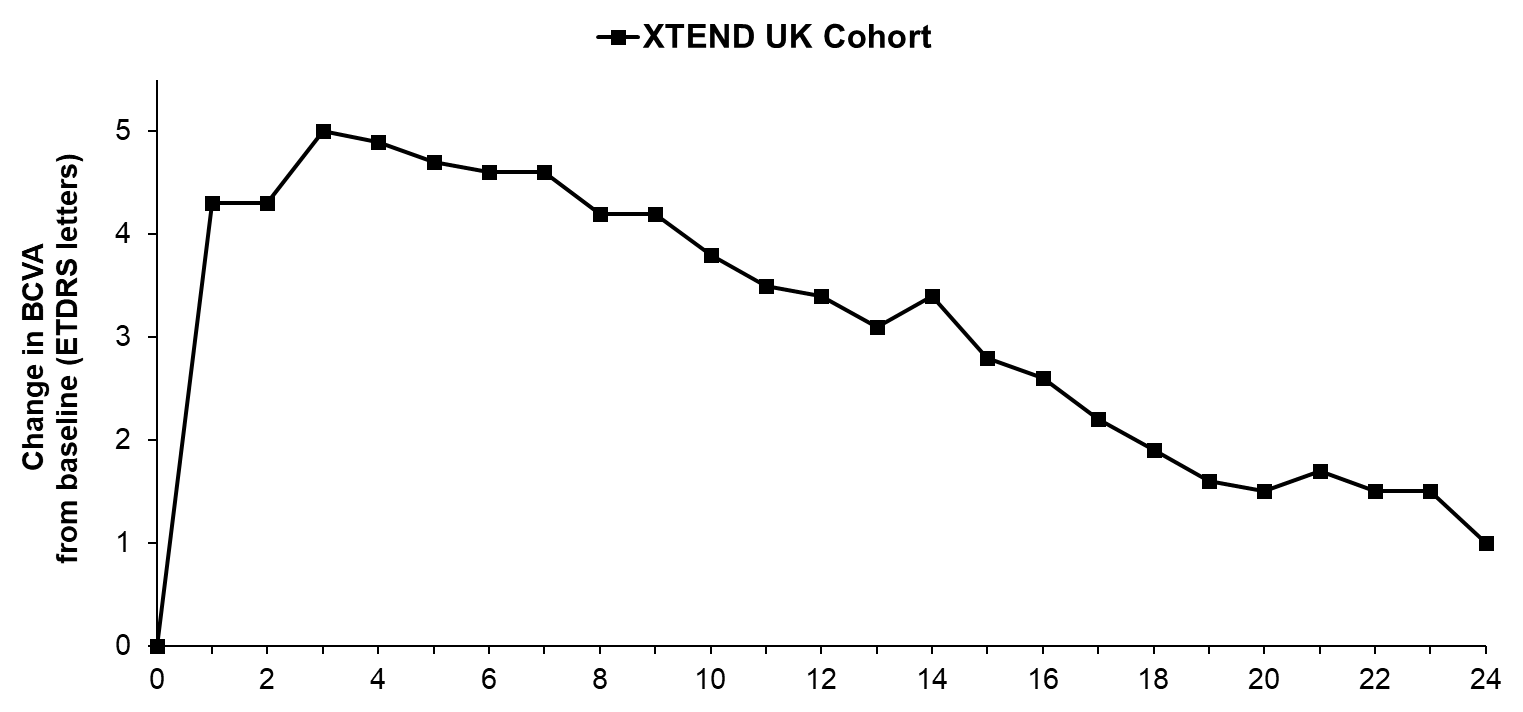


FAS, LOCF. Mean BCVA change data reported here are based on the nearest BCVA assessments within the monthly +/-15-day visit windows. Data for completers only available at Month 12 and Month 24 (shown in Table 2). BCVA, best-corrected visual acuity; EDTRS, Early Treatment Diabetic Retinopathy Study; FAS, full analysis set; LOCF, last observation carried forward.

**(b)**


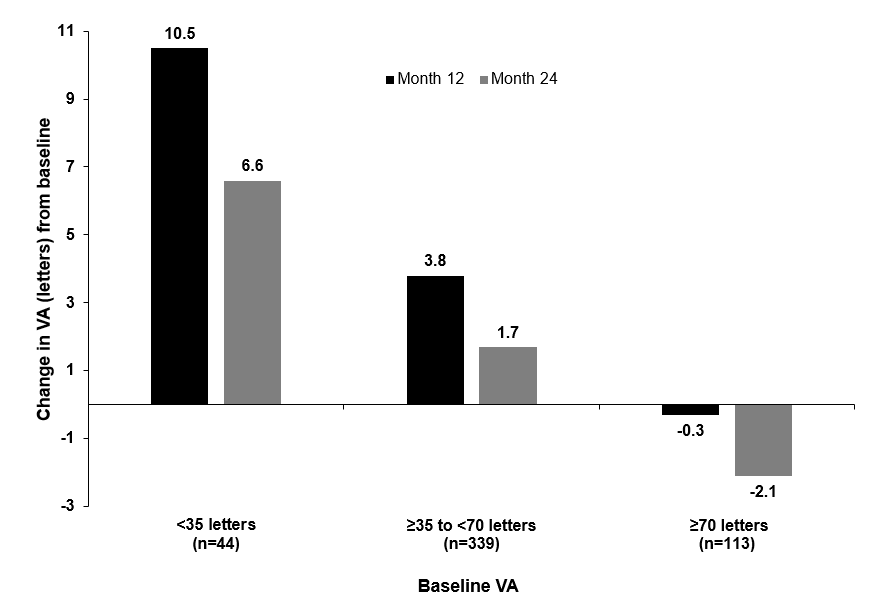
FAS, LOCF. FAS, full analysis set; LOCF, last observation carried forward; VA, visual acuity.

**Supplementary Table 2: Mean change in CST measured by OCT (μm) from baseline to Month 12 and Month 24 in patients with treatment-naïve nAMD who received IVT-AFL in routine clinical practice.**
FAS, LOCF. Data are mean±SD and mean (95% CI) unless otherwise stated. The mean CST change data are based on the nearest CST assessment within the ±60-day visit window of 360 days for Month 12 and 720 days for Month 24. CI, confidence interval; CST, central subfield thickness; FAS, full analysis set; LOCF, last observation carried forward; OCT, optical coherence tomography; nAMD, neovascular age-related macular degeneration; SD, standard deviation.

|  | **XTEND UK Cohort N=496** |
| --- | --- |
| **Baseline**  Mean CST, μm  n  **Month 12**  Mean CST, μm  Change from BL  n  **Month 24**  Mean CST, μm  Change from BL  n | 395±143  344  291±111  –105 (–121, –89)  393  285±112  –105 (–122, –88)  423 |

**Supplementary Fig. 2: Mean change in CST measured by OCT (μm) from baseline to Month 12 and Month 24 in patients with treatment-naïve nAMD who received IVT-AFL in routine clinical practice.**
FAS, LOCF. The mean CST change data are based on the nearest CST assessment within the ±60-day visit window of 360 days for Month 12 and 720 days for Month 24. CI, confidence interval; CST, central subfield thickness; FAS, full analysis set; LOCF, last observation carried forward; OCT, optical coherence tomography; nAMD, neovascular age-related macular degeneration; SD, standard deviation.


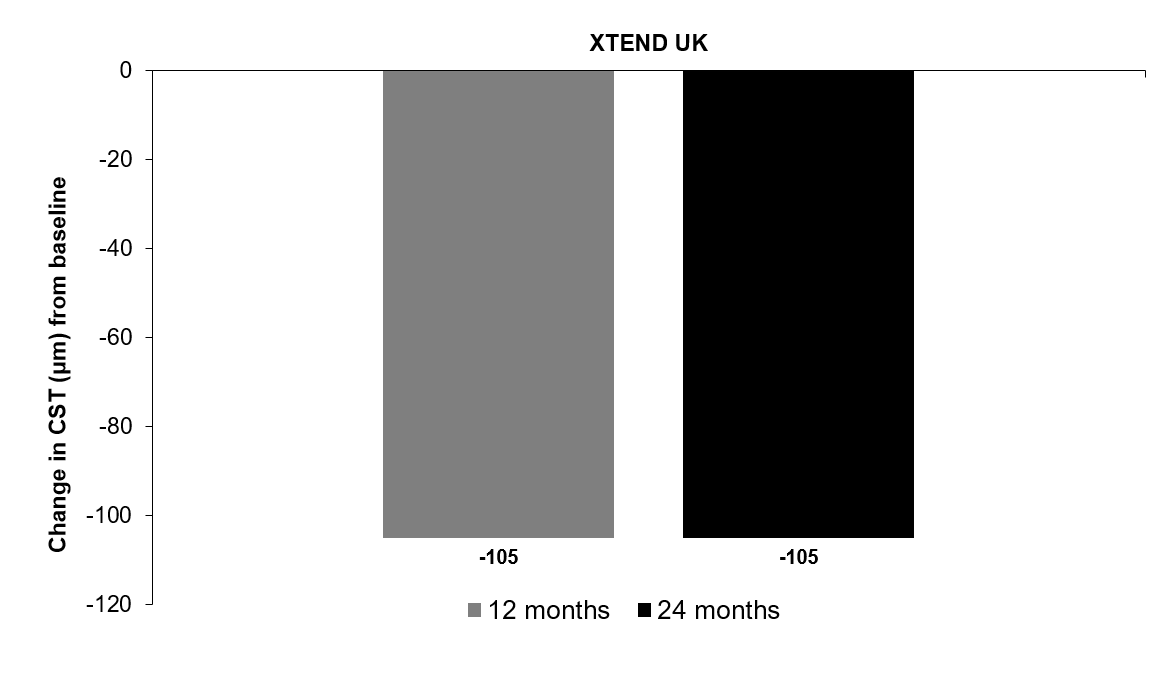


**Supplementary Table 3:** **Safety overview cumulative up to 24 months (SAS).**

| **Number of patients (%)** | **XTEND UK Cohort (N=518)** |
| --- | --- |
| **Any AE** | 220 (42.5) |
| **Any TEAE**  Any ocular TEAE  Any non-ocular TEAE  Any ocular TEAE in the study eye | 192 (37.1)  130 (25.1)  99 (19.1)  85 (16.4) |
| **Most common ocular TEAEs in the study eye (≥1%)^a^**  **Cataract**  Blepharitis  Cataract nuclear  Conjunctival haemorrhage  Visual impairment | 17 (3.3)  9 (1.7)  7 (1.4)  6 (1.2)  5 (1.0) |
| **Any intraocular inflammation in the study eye**  Eye infection  Endophthalmitis  Iridocyclitis | 5 (1.0)  3 (0.6)  1 (0.2)  1 (0.2) |
| **Any serious TEAEs**  Serious ocular TEAEs  Any serious drug-related TEAE  Any serious drug-related ocular TEAE  Any serious drug-related non-ocular TEAE  Any serious TEAE causally related to IVT-AFL injection procedure^b^ | 80 (15.4)  27 (5.2)  7 (1.4)  4 (0.8)  3 (0.6)  1 (0.2) |
| **Most common serious ocular TEAEs (≥2%)**  nAMD^c^ | **12 (2.3)** |
| **Death**  Treatment-emergent  Non-treatment-emergent | 4 (0.8)  3 (0.6)  1 (0.2) |

^a^11 (2.1%) ocular TEAEs not yet coded. ^b^Injection-site inflammation. ^c^Worsening of nAMD, as is defined in the study protocol. AE, adverse event; IVT-AFL, intravitreal aflibercept; nAMD, neovascular age-related macular degeneration; SAS, safety analysis set; TEAE, treatment-emergent adverse event.

**REFERENCES**

1. Korobelnik JF, Chaudhary V, Mitchell P, Kang SW, Tadayoni R, Allmeier H, et al. XTEND: Two-Year Results from a Global Observational Study Investigating Proactive Dosing of Intravitreal Aflibercept in Neovascular Age-Related Macular Degeneration. Ophthalmol Ther. 2024;13(3):725-38.
